# Carotid plaque number and length as predictors of coronary heart disease in patients with early-stage chronic kidney disease

**DOI:** 10.3389/fcvm.2026.1793887

**Published:** 2026-04-10

**Authors:** Cui-Fen Hu, Qing-Yin Fu, Yan-Fei Zhang, Qi-Ping Liu, De-Bin Yang, Bin Hu

**Affiliations:** 1 Department of Ultrasound in Medicine, Minhang Hospital, Fudan University, Shanghai, China; 2 Department of Ultrasound in Medicine, Jiading District Central Hospital Afﬁliated Shanghai University of Medicine&Health Sciences, Shanghai, China

**Keywords:** carotid ultrasound parameter, carotid plaque number, maximum carotid plaque length, chronic kidney disease, coronary heart disease

## Abstract

**Background:**

Chronic kidney disease (CKD) is an established risk factor for coronary heart disease (CHD). Current CHD diagnostics—coronary angiography or CTA—require contrast agents that may worsen renal function or induce contrast-induced nephropathy. A non-contrast, non-invasive, and relatively accurate method is urgently needed to predict CHD in CKD patients.

**Methods:**

In this retrospective analysis (2020.04–2024.10), patients with CKD were classified into stable CHD or non-CHD groups based on coronary CTA. Carotid ultrasound parameters were collected. Propensity score matching (PSM) controlled for confounders. Logistic regression identified CHD risk factors, and ROC analysis evaluated predictive performance.

**Results:**

A total of 377 patients with renal insufficiency were enrolled, most of whom were in CKD stage 2. Among them, 144 patients had stable CHD. After matching, 114 patients per group were analyzed. Carotid plaque number (OR = 2.074, 95% CI: 1.243–3.460, *p* = 0.005) and maximum plaque length (OR = 1.165, 95% CI: 1.073–1.265, *p* < 0.001) were significant predictors. AUC values were 0.593 (*p* = 0.032) for plaque number, 0.696 (*p* < 0.001) for plaque length, and 0.716 (*p* < 0.001) for the combined model, with optimal cutoffs of 2.50 plaques and 8.75 mm.

**Conclusion:**

Carotid plaque number and length are useful predictors of CHD in CKD patients, especially in the early stage. Carotid ultrasound may serve as a non-invasive tool for early detection and improved long-term outcomes in this high-risk population.

## 1 Introduction

Cardiovascular diseases are the leading cause of death globally, with ischemic cardiomyopathy having the highest incidence of cardiovascular death (1). Coronary heart disease (CHD) is the main cause of ischemic cardiomyopathy. Therefore, early intervention for CHD is crucial for reducing cardiovascular mortality. The pathogenesis of CHD is multifactorial. In addition to the well-established traditional risk factors (2) (such as advanced age, obesity, smoking, abnormal glucose tolerance or diabetes, hypertension, and dyslipidemia characterized by elevated low-density lipoprotein cholesterol), recent studies have further identified several emerging independent risk factors. For example, mental and psychological diseases (3), elevated lipoprotein(a) levels (4), and gut microbiota disorders (5) play important roles in inducing or accelerating the formation of coronary atherosclerotic plaques. In addition to the above factors, chronic renal insufficiency (CRI) is one of the important etiologies for the occurrence and development of CHD. Moreover, it is a key factor leading to the complexity of clinical management and an increased risk of death in patients with CRI complicated by CHD.

Chronic kidney disease (CKD)-related CRI remains a global health burden (6). According to KDIGO guidelines, CKD is staged from G1 to G5 based on estimated glomerular filtration rate (eGFR) (7). Evidence indicates that CRI is strongly associated with CHD risk: eGFR below 90 mL/min/1.73 m^2^ significantly elevates CHD risk, an eGFR below 60 mL/min/1.73 m^2^ (stage G3a and beyond) exhibits a robust linear relationship with increased CHD incidence and all-cause mortality (8, 9). Notably, patients in stages G3b–G4 face a cardiovascular mortality risk more than 2–3 times higher than those with normal renal function (8, 9). Thus, CKD constitutes an independent risk factor for CHD and promotes atherosclerosis even at early stages (e.g., G2). Early detection and management of CHD in CKD patients are critical for reducing cardiovascular mortality in this population.

The diagnosis of CHD mainly relies on coronary CTA or angiography. However, the iodine-containing contrast agents necessary for these examinations pose significant risks to patients with CKD, which can lead to contrast-induced nephropathy (CIN), exacerbate renal function deterioration, and increase mortality (10, 11). Therefore, there is an urgent clinical need to develop an effective contrast-free method to evaluate the CHD status in CKD patients. Carotid ultrasound examination, with its advantages of no radiation exposure, no need for contrast agents, safety, simplicity, repeatability, and real-time imaging, has been widely used in the diagnosis of vascular diseases. Numerous studies have confirmed the predictive value of carotid plaque characteristics for the risk of CHD in the general population (12). However, there is insufficient evidence regarding its predictive efficacy for CHD in the special population of CKD patients. For this reason, this study adopted a retrospective case-control design, aiming to evaluate the predictive value of carotid ultrasound plaque parameters for CHD in CKD patients by comparing CKD patients with stable CHD and CKD controls without CHD, in order to provide evidence-based support for the early non-invasive diagnosis and intervention of CHD in CKD patients.

## 2 Materials and methods

### 2.1 Study design and patients

This study was a retrospective case-control investigation. Consecutive patients aged 18 years or older who were hospitalized at Minhang Hospital, Fudan University between 2020.04 and 2024.10 were selected. Based on predefined inclusion and exclusion criteria, the case group comprised patients with CKD complicated by CHD, while the control group consisted of patients with CKD without CHD. The case group included 144 participants, and the control group included 233 participants. Inclusion criteria were: age ≥18 years, diagnosis of CKD, completion of both coronary CTA and carotid artery ultrasound during hospitalization. Exclusion criteria consisted of: clinical diagnosis of acute coronary syndrome during hospitalization, history of prior myocardial infarction, presence of autoimmune diseases, severe hepatic insufficiency, other severe comorbidities such as malignancy with a life expectancy of less than one-year or significant valvular heart disease.

The study was conducted in accordance with the ethical principles of the Declaration of Helsinki (2013 revision). The study protocol was approved by the Ethics Committee of Minhang Hospital, Fudan University (approval No: 2025-103-01K) and individual consent for this retrospective analysis was waived.

### 2.2 Definition of CKD and CHD

CKD is defined as the presence of structural or functional renal abnormalities that persist for at least three months. The condition is classified into five stages according to estimated glomerular filtration rate (eGFR) thresholds, as follows: stage G1 (normal or high: eGFR ≥90 mL/min/1.73 m^2^), G2 (mildly reduced: 60–89), G3a (mildly to moderately reduced: 45–59), G3b (moderately to severely reduced: 30–44), G4 (severely reduced: 15–29), and G5 (kidney failure: <15) (7). All participants included in this study had a confirmed diagnosis of CKD.

In this study, CHD was defined as the presence of coronary artery stenosis exceeding 50%, as assessed by coronary CTA, attributable to atherosclerotic plaque formation. All coronary CTA scans were quantitatively analyzed by experienced radiologists in adherence to established guidelines. Stenosis severity was determined using computer-assisted quantitative software rather than visual estimation alone. Moreover, a standardized protocol was uniformly applied to evaluate all coronary segments. Regarding plaque characterization, components such as vulnerability and calcification were also assessed. All enrolled patients met the diagnostic criteria for chronic coronary syndromes (13). Consistent with contemporary clinical guidelines, individuals with a history of acute coronary syndrome or myocardial infarction were excluded from the cohort (14).

### 2.3 Carotid artery ultrasound examination and parameters

Prior to examination, all patients rested for 5–10 min to stabilize respiratory and heart rates. Subjects were positioned supine with a soft pillow supporting the neck and the head extended to optimize exposure of the carotid region. Neck accessories were removed, and patients were instructed to maintain quiet breathing and refrain from swallowing during the scan.

Examinations were conducted using the Canon Aplio 500, Aplio i900, and other ultrasound systems with a linear array probe set at 5–14 MHz. Longitudinal scans were performed along the carotid arteries to identify atherosclerotic plaques and stenosis. When present, plaque characteristics including morphology, length, thickness, echogenicity, and homogeneity were documented, along with the degree of stenosis. Plaque count and carotid intima–media thickness (cIMT) were recorded. Color Doppler imaging was used to measure the resistive index (RI).

Ultrasound parameters were defined as follows: the number of plaques was treated as a discrete numeric variable, capped at 3—meaning any patient with three or more plaques was assigned a value of 3; plaque thickness and length corresponded to the largest identified plaque; cIMT and RI values were taken as the maximum from bilateral measurements. Plaque vulnerability was scored for each side according to the Chinese expert consensus on ultrasound evaluation of vulnerable carotid plaque (15) and related literature (16) (see Table 1), with the total score representing the sum of both sides.

**Table 1:** Carotid Plaque Vulnerability Assessment Reference.

Standard	Characteristics	Scoring
Morphology	Smooth plaque surface, intact fibrous cap	0
	Rough plaque surface, incomplete fibrous cap	1
	Ulcerated plaque	2
Homogeneity	Homogeneous echo	0
	Heterogeneous echo	1
Echo characteristics	Strong echo	0
	Mixed echo	1
	Low echo	2
Degree of vascular stenosis	Mild: Stenosis < 50%	0
	Moderate: Stenosis 50%–69%	1
	Severe: Stenosis 70%–99%	2

The total plaque vulnerability score was calculated as the sum of the vulnerability scores from the largest plaques in the bilateral carotid arteries.

### 2.4 Blood Biochemical examination

Venous blood samples were collected from both case and control subjects upon admission. Laboratory analyses included complete blood count, biochemical profiling, lipid panel, blood glucose levels, and coagulation tests. All assays were conducted in strict accordance with the manufacturer's instructions provided with the corresponding kits.

### 2.5 Statistical analysis

Categorical variables were expressed as percentages and compared using *χ*^2^, Fisher's exact, or Cochran–Mantel–Haenszel tests, as appropriate. Continuous variables were presented as mean ± SD or median (the 25th percentile (P25) and the 75th percentile (P75)), based on distribution normality assessed by the Shapiro–Wilk test. Group comparisons were performed using independent *t*-tests for normally distributed data and Wilcoxon rank-sum tests for non-normal data.

To mitigate potential selection bias and confounding effects, Propensity score matching (PSM) was applied. Scores were derived from a logistic regression model that included demographic (age, sex, height, weight, BMI, blood pressure, heart rate, smoking, drinking) and clinical covariates (diabetes, hypertension, dyslipidemia, cerebral ischemic stroke, tumor). Nearest neighbor matching with a caliper of 0.02.

Significant variables identified in paired comparisons were further analyzed using logistic regression to estimate odds ratio (OR) with 95% confidence intervals (CIs). Predictive performance was evaluated using receiver operating characteristic (ROC) curve analysis. *p* < 0.05 was considered statistically significant. All analyses were conducted using SPSS version 26.0.

## 3 Results

A total of 377 patients were ultimately enrolled in this study based on the predefined inclusion and exclusion criteria. Among them, 144 patients were assigned to the case group (comprising individuals with both CHD and CKD), while the remaining 233 patients were designated as the control group (consisting of those with CKD but without CHD). Following PSM, 114 well-balanced cohort pairs were successfully matched.

### 3.1 Comparison of baseline characteristics before and after PSM

Prior to PSM, significant baseline discrepancies were observed between the cohorts across several parameters, including age, sex distribution, body weight, and histories of diabetes, hypertension, and stroke. Following matching, these covariates were well-balanced, with no statistically significant differences remaining between the groups (Table 2). The standardized mean difference (SMD) further confirmed adequate covariate balance after matching, with nearly all variables showing absolute SMD values below 0.1 (Table 2).

**Table 2:** Baseline demographic and clinical variables before and after PSM.

Variables	Unmatched analysis				Propensity score matched analysis			
	CHD	No CHD	*P* value	SMD	CHD	No CHD	*P* value	SMD
	(*n* = 144)	(*n* = 233)			(*n* = 114)	(*n* = 114)		
Age (yr)	74.00 (68.00–81.00)	71.00 (65.00–76.00)	**<0**.**001**	0.386	72.00 (68.00–80.00)	72.00 (67.00–78.00)	0.426	0.078
Sex (Male, %)	61.1	39.9	**<0**.**001**	0.434	56.1	60.5	0.502	−0.089
Height (m)	1.65 (1.60–1.70)	1.63 (1.56–1.70)	0.14	0.272	1.65 (1.60–1.70)	1.66 (1.60–1.72)	0.211	−0.145
Weight (Kg)	69.00 (61.63–75.00)	64.00 (55.00–72.00)	**0**.**001**	0.294	68.00 (61.00–75.00)	67.25 (59.38–75.00)	0.982	−0.014
BMI (Kg/m^2^)	24.78 (23.34–26.62)	24.03 (22.05–26.35)	0.590	0.170	24.98 (23.14–26.82)	25.03 (22.25–26.84)	0.745	0.073
SBP (mmHg)	131.00 (120.00–145.00)	130 (120–140)	0.470	0.170	130.00 (120.00–142.50)	130.00 (120.00–147.50)	0.994	−0.053
DBP (mmHg)	79.50 (70.00–83.50)	78 (70–80)	0.284	0.075	78.00 (70.00–80.00)	79.00 (70.00–80.00)	0.928	−0.070
HR (bpm)	78.00 (72.00–84.00)	80.00 (72.00–86.00)	0.257	−0.112	79.00 (72.00–84.00)	79.00 (72.00–84.00)	0.587	0.041
Smoking%	14.6	12.9	0.638	0.050	14.0	17.5	0.468	−0.096
Drinking%	5.6	6.9	0.612	−0.054	5.3	7.9	0.423	−0.106
Diabetes%	36.8	22.3	**0**.**002**	0.322	30.7	33.3	0.670	−0.056
Hypertension%	75.7	57.1	**<0**.**001**	0.402	73.7	70.2	0.555	0.078
Dyslipidemia%	10.4	13.3	0.405	−0.089	9.6	7.0	0.472	0.095
Cerebral ischemic stroke%	12.5	5.2	**0**.**010**	0.261	9.6	8.8	0.819	0.030
Tumor%	6.3	5.6	0.787	0.028	7.0	7.0	1.000	0.000

CHD, coronary heart disease; BMI, body mass index; SBP, systolic blood pressure; DBP, diastolic blood pressure; HR, heart rate.

Bold indicates statistical differences (*P* < 0.05).

### 3.2 Comparison of routine hematological and biochemical parameters before and after PSM

The overall statistical profiles of routine hematological and biochemical parameters for both cohorts were presented in Table 3. Prior to PSM, univariate analysis revealed statistically significant differences in 13 parameters between the groups. Following PSM, only thrombin time and fibrinogen demonstrated persistent intergroup differences.

**Table 3:** Blood routine and biochemical markers before and after PSM.

Variables	Unmatched analysis			Propensity score matched analysis		
	CHD	No CHD	*P* value	CHD	No CHD	*P* value
	(*n* = 144)	(*n* = 233)		(*n* = 114)	(*n* = 114)	
Hemoglobin (g/L)	130.00 (119.25–139.75	127.00 (119.00–138.50)	0.322	130.00 (120.75–144.00)	133.00 (122.00–142.25)	0.508
Red Blood Cell (10^12/L)	4.27 ± 0.57	4.19 ± 0.50	0.144	4.26 (3.93–4.68)	4.29 (3.97–4.58)	0.950
Platelet (10^9/L)	190.50 (156.00–232.75)	205.00 (165.00–240.00)	0.087	191.50 (155.75–233.50)	195.50 (157.75–234.25)	0.766
White Blood Cell (10^12/L)	6.32 (5.22–7.48)	5.88 (4.83–6.94)	**0**.**018**	6.29 (5.17–7.55)	6.22 (5.25–7.27)	0.689
Neutrophil%	60.61 ± 10.29	57.97 ± 10.11	0.980	59.61 ± 10.44	59.63 ± 10.74	0.580
Lymphocyte%	27.77 ± 9.37	30.81 ± 9.17	0.848	28.80 ± 9.46	28.86 ± 9.50	0.770
Monocyte%	7.80 (6.80–8.98)	7.70 (6.50–9.20)	0.708	7.80 (6.78–8.63)	7.80 (6.78–9.00)	0.605
Neutrophil (10^9/L)	3.86 (2.91–4.83)	3.41 (2.61–4.25)	**0**.**004**	3.73 (2.83–4.80)	3.62 (2.82–4.58)	0.785
Lymphocyte (10^9/L)	1.63 (1.36–2.04)	1.77 (1.37–2.15)	0.260	1.69 (1.41–2.10)	1.69 (1.35–2.07)	0.768
Monocyte (10^9/L)	0.50 (0.40–0.59)	0.46 (0.38–0.56)	**0**.**037**	0.49 (0.38–0.59)	0.500 (0.400–0.59)	0.785
Kalium (mmol/L）	3.90 (3.60–4.10)	3.90 (3.70–4.20)	0.645	3.90 (3.60–4.10)	3.98 (3.70–4.10)	0.455
Natrium (mmol/L）	141.00 (140.00–143.00)	142.00 (140.00–143.50)	**0**.**027**	141.00 (140.00–143.00)	142.00 (140.00–143.00)	0.854
Chlorinum (mmol/L）	104.00 (102.00–106.00)	104.00 (102.00–106.00)	0.147	104.00 (102.00–106.00)	103.00 (101.00–105.00)	0.163
Glucose (mmol/L）	5.80 (5.10–6.875)	5.50 (4.90–6.30)	**0**.**011**	5.60 (5.08–6.60)	5.65 (5.00–6.80)	0.898
Glycated Hemoglobin %	6.60 (6.20–7.70)	6.20 (5.90–7.10)	**<0**.**001**	6.50 (6.10–7.50)	6.60 (5.90–7.53)	0.487
Alanine Aminotransferase (U/L)	14.00 (10.00–21.00)	15.00 (11.00–23.00)	0.378	14.00 (11.00–23.00)	16.00 (11.75–24.25)	0.392
Aspartic Aminotransferase (U/L)	15.50 (13.00–21.00)	17.00 (14.00–21.00)	0.156	16.00 (13.75–23.00)	17.00 (14.00–21.00)	0.367
Alkaline Phosphatase (mmol/L）	69.00 (58.00–82.75)	69.00 (58.00–82.00)	0.877	69.00 (58.00–83.50)	69.00 (55.00–80.25)	0.545
Lactate Dehydrogenase (U/L)	159.00 (141.50–179.00)	159.00 (141.50–181.00)	0.967	159.00 (140.50–179.25)	158.50 (141.75–174.50)	0.769
Creatinine (umol/L)	84.00 (71.00–98.00)	79.00 (68.00–95.50)	0.093	81.00 (68.75–96.25)	85.00 (72.00–103.00)	0.052
Blood urea nitrogen (mmol/L)	5.70 (4.50–6.88)	5.20 (4.50–6.40)	0.276	5.40 (4.40–6.63)	5.40 (4.58–6.93)	0.253
eGFR	72.28 (58.77–83.89)	75.69 (61.00–84.38)	0.345	75.57 (61.71–85.36)	73.47 (56.58–81.96)	0.099
Uric Acid (*μ*mol/L)	353.00 (280.25–410.50)	341.00 (270.50–396.00)	0.160	352.50 (282.50–405.00)	370.00 (292.50–429.25)	0.200
Triglyceride (mmol/L)	1.23 (0.95–1.75)	1.34 (0.98–1.91)	0.201	1.24 (0.95–1.75)	1.39 (0.99–1.96)	0.149
Total cholesterol (mmol/L)	3.95 (3.18–4.59)	4.42 (3.53–5.07)	**0**.**001**	3.94 (3.28–4.65)	4.03 (3.29–4.96)	0.445
High-density lipoprotein (mmol/L)	1.07 (0.91–1.24）	1.15 (0.98–1.37)	**0**.**001**	1.08 (0.92–1.25)	1.06 (0.96–1.27)	0.853
Low-density lipoprotein (mmol/L)	2.35 (1.77–2.98)	2.73 (1.96–3.46)	**0**.**003**	2.34 (1.85–2.94)	2.66 (1.79–3.35)	0.181
Lipoprotein *α*	129.50 (64.50–420.00)	148.00 (54.50–296.50)	0.491	135.00 (66.75–414.00)	134.00 (57.75–236.00)	0.216
Prothrombin time (s)	11.80 (11.10–12.30)	11.50 (11.10–12.00)	**0**.**045**	11.70 (10.95–12.25)	11.60 (11.05–12.00)	0.314
International Normalized Ratio	1.03 (0.96–1.07)	1.00 (0.96–1.04)	**0**.**040**	1.02 (0.95–1.07)	1.01 (0.96–1.05)	0.310
Activated Partial Thromboplastin (s)	27.6 (24.40–30.10)	26.95 (24.43–29.30)	0.519	27.60 (24.65–29.90)	27.30 (24.50–29.15)	0.741
Thrombin Time (s)	17.20 (16.40–17.80)	17.30 (16.60–18.10)	0.067	17.20 (16.40–17.80)	17.60 (16.85–18.30)	**0**.**005**
Fibrinogen (g/L)	3.01 (2.45–3.46)	2.73 (2.39–3.21)	**0**.**011**	3.03 (2.42–3.49)	2.67 (2.27–3.17)	**0**.**004**
D-dimer (mg/L）	0.47 (0.23–0.78)	0.31 (0.18–0.61)	**0**.**002**	0.42 (0.22–0.72)	0.31 (0.18–0.66）	0.197

CHD, coronary heart disease; eGFR, estimated glomerular filtration rate.

Bold indicates statistical differences (*P* < 0.05).

### 3.3 Comparison of baseline characteristics in patients with carotid plaque

To further evaluate the predictive value of carotid plaque parameters for stable CHD in patients with CKD, we conducted a PSM analysis. After matching, 81 out of 114 control patients and 97 out of 114 patients in the case group had identifiable carotid plaques. Subsequent comparative analysis of these plaque-bearing subgroups revealed no statistically significant differences in baseline demographic or clinical characteristics. However, a significant intergroup difference was observed in the prevalence of carotid plaques (*p* = 0.010) (Table 4).

**Table 4:** Baseline demographic and clinical variables in patients with CKD and carotid artery plaques.

Variables	CHD (*n* = 97)	No CHD (*n* = 81)	*P* value
Age (yr)	73.33 ± 9.81	72.74 ± 9.50	0.719
Sex (Male, %)	61.9	60.5	0.853
Height (m)	1.65 (1.60–1.70)	1.65 (1.59–1.72)	0.550
Weight (Kg)	70.00 (60.00–74.50)	65.00 (59.25–75)	0.843
BMI (Kg/m^2^)	24.68 (22.90–26.32)	25.08 (22.13–26.98)	0.905
SBP (mmHg)	130.00 (120.00–144.50)	130.00 (120.00–148.50)	0.919
DBP (mmHg)	76.00 (70.00–80.00)	78.00 (70.00–80.00)	0.946
HR (bpm)	80.00 (72.00–84.00)	80.00 (72.50–84.00)	0.900
Smoking%	14.4	14.8	0.943
Drinking%	6.2	8.6	0.531
Diabetes%	35.1	30.9	0.555
Hypertension%	74.2	70.4	0.566
Dyslipidemia%	8.2	8.6	0.925
Cerebral ischemic stroke%	10.3	9.9	0.924
Tumor%	7.2	3.7	0.311
Carotid artery plaque (%)	85.09	71.05	**0**.**010**

CKD, chronic kidney disease; CHD, coronary heart disease; BMI, body mass index; SBP, systolic blood pressure; DBP, diastolic blood pressure; HR, heart rate.

Bold indicates statistical differences (*P* < 0.05).

### 3.4 Routine hematological and biochemical parameters in patients with carotid plaque

As presented in Table 5, the biochemical profiles of the two patient groups with carotid plaque were compared. Univariate analysis revealed statistically significant intergroup differences in the following parameters: estimated glomerular filtration rate (eGFR), triglyceride levels, thrombin time, and fibrinogen concentration.

**Table 5:** Blood routine and biochemical markers in patients with CKD and carotid artery plaques.

Variables	CHD (*n* = 97)	No CHD (*n* = 81)	*P* value
Hemoglobin (g/L)	131.40 ± 20.82	130.51 ± 17.30	0.644
Red Blood Cell (10^12/L)	4.27 ± 0.55	4.22 ± 0.54	0.565
Platelet (10^9/L)	198.75 ± 63.84	201.69 ± 60.49	0.837
White Blood Cell (10^12/L)	6.27 (5.23–7.64)	6.29 (5.47–7.30)	0.955
Neutrophil%	60.44 ± 10.33	60.29 ± 11.10	0.529
Lymphocyte%	27.91 ± 9.25	28.18 ± 9.93	0.516
Monocyte%	7.80 (6.80–8.80)	7.80 (6.85–8.90)	0.747
Neutrophil (10^9/L)	3.74 (2.89–4.84)	3.75 (3.03–4.78)	0.976
Lymphocyte (10^9/L)	1.66 (1.41–2.06)	1.68 (1.30–2.13)	0.993
Monocyte (10^9/L)	0.49 (0.37–0.61)	0.51 (0.42–0.59)	0.548
Kalium (mmol/L）	3.90 (3.60–4.15)	3.90 (3.70–4.10)	0.825
Natrium (mmol/L）	141.00 (140.00–143.00)	142.00 (140.00–143.00)	0.429
Chlorinum (mmol/L）	104.00 (102.00–105.50)	103.00 (101.00–105.00)	0.397
Glucose (mmol/L）	5.60 (5.10–6.60)	5.60 (4.95–6.65)	0.700
Glycated Hemoglobin %	6.50 (6.20–7.50)	6.50 (5.90–7.30)	0.166
Alanine Aminotransferase (U/L)	14.00 (10.50–21.00)	15.00 (10.00–25.50)	0.478
Aspartic Aminotransferase (U/L)	16.00 (13.00–20.50)	18.00 (14.00–22.50)	0.139
Alkaline Phosphatase (mmol/L）	69.00 (58.00–82.00)	67.00 (55.50–78.50)	0.528
Lactate Dehydrogenase (U/L)	160.00 (142.50.50–181.50)	160.00 (144.50–177.00)	0.884
Creatinine (umol/L)	84.00 (71.00–98.00)	93.00 (72.00–110.50)	0.054
Blood urea nitrogen (mmol/L)	5.70 (4.55–6.75)	5.80 (4.70–7.25)	0.260
eGFR	73.78 (60.70–84.68)	68.20 (53.51–80.21)	**0**.**032**
Uric Acid (μmol/L)	351.26 ± 90.08	376.98 ± 119.55	0.057
Triglyceride (mmol/L)	1.23 (0.95–1.67)	1.40 (1.00–2.11)	**0**.**049**
Total cholesterol (mmol/L)	3.88 (3.25–4.56)	4.00 (3.42–4.98)	0.215
High-density lipoprotein (mmol/L)	1.08 (0.93–1.25)	1.04 (0.95–1.26)	0.706
Low-density lipoprotein (mmol/L)	2.34 (1.85–3.06)	2.67 (1.84–3.44)	0.252
Lipoprotein α	153.00 (66.50–417.00)	125.00 (57.50–228.50)	0.191
Prothrombin time (s)	11.80 (10.90–12.30)	11.60 (11.10–12.00)	0.319
International Normalized Ratio	1.03 (0.94–1.07)	1.01 (0.96–1.04)	0.305
Activated Partial Thromboplastin (s)	27.60 (24.78–29.95)	26.95 (24.50–28.90)	0.647
Thrombin Time (s)	17.20 (16.43–17.80)	17.70 (16.80–18.30)	**0**.**024**
Fibrinogen (g/L)	3.02 (2.43–3.53)	2.68 (2.24–3.20)	**0**.**014**
D-dimer (mg/L）	0.44 (0.23–0.72)	0.32 (0.19–0.74)	0.245

CKD, chronic kidney disease; CHD, coronary heart disease; eGFR, estimated glomerular filtration rate.

Bold indicates statistical differences (*P* < 0.05).

### 3.5 Echocardiographic and carotid ultrasound parameters in patients with carotid plaque

Tables 6, 7 summarize the echocardiographic and carotid ultrasound parameters for both patient groups, along with comparative analyses. No statistically significant differences were observed in any echocardiographic parameters between the groups.

**Table 6:** Echocardiogram findings in patients with CKD and carotid artery plaques.

Variables	CHD (*n* = 97)	No CHD (*n* = 81)	*P* value
LVEF (%)	63.00 (61.00–65.00)	64.00 (61.00–65.00)	0.383
Left atrial diameter (mm)	41.00 (38.00–42.00)	41.00 (38.00–43.00)	0.959
Left ventricular end-systolic diameter (mm）	29.00 (27.00–31.00)	29.00 (27.00–31.00)	0.975
Left ventricular end-diastolic diameter (mm）	45.00 (43.00–48.00)	46.00 (44.00–49.00)	0.234
Left ventricular posterior wall diameter (mm)	10.00 (9.00–10.00)	10.00 (9.00–10.00)	0.344
Interventricular septal thickness (mm)	10.00 (9.00–11.00)	10.00 (10.00–11.00)	0.548

CKD, chronic kidney disease; LVEF, left ventricular ejection fraction.

**Table 7:** Carotid artery ultrasound findings in patients with CKD and carotid artery plaques.

Variables	CHD (*n* = 97)	No CHD (*n* = 81)	*P* value
Carotid artery plaque number	3.00 (3.00–3.00)	3.00 (2.00–3.00)	**0**.**002**
Max IMT (mm）	0.80 (0.70–0.80)	0.80 (0.70–0.90)	0.132
Max Carotid artery plaque length (mm)	9.00 (6.50–13.00)	6.30 (4.75–8.50)	**<0**.**001**
Max Carotid artery plaque thickness (mm)	2.20 (1.75–2.70)	1.80 (1.60–2.30)	**0**.**005**
Max Common carotid artery RI	0.80 ± 0.00543	0.79 ± 0.00625	0.791
Max Internal carotid artery RI	0.70 ± 0.00678	0.68 ± 0.00654	0.211
Vulnerable plaque score	2.00 (0.00–4.00)	1.00 (0.00–2.00)	**0**.**001**

CKD, chronic kidney disease; IMT, carotid intima-media thickness; RI, Resistive Index.

Bold indicates statistical differences (*P* < 0.05).

In contrast, significant differences were detected in carotid ultrasound measures, including the number of plaques, maximum plaque length and thickness, and plaque vulnerability score. All of these carotid plaque parameters were significantly higher in the case group compared to the control group.

### 3.6 OR for CHD in CKD patients with carotid plaque using logistic regression analysis

The multivariate logistic regression model incorporated all statistically significant biochemical markers and the complete set of carotid plaque ultrasound parameters. Risk factors with significant differences for CHD in CKD patients included carotid plaque number (OR = 2.074, *P* = 0.005, 95% CI: 1.243–3.460), maximum carotid plaque length (OR = 1.165, *P* < 0.001, 95% CI: 1.073–1.265), see Table 8.

**Table 8:** OR for CKD-related CHD using logistic regression analysis.

Variables	B value	SE	Odds ratio	95% CI	*P* value
Carotid artery plaque number	0.729	0.261	2.074	1.243–3.460	0.005
Max Carotid artery plaque length (mm)	0.153	0.042	1.165	1.073–1.265	<0.001

CKD, chronic kidney disease; CHD, coronary heart disease; SE, standard error; CI, confidence interval.

### 3.7 ROC analysis for predicting CHD in CKD patients

**Figure 1:**
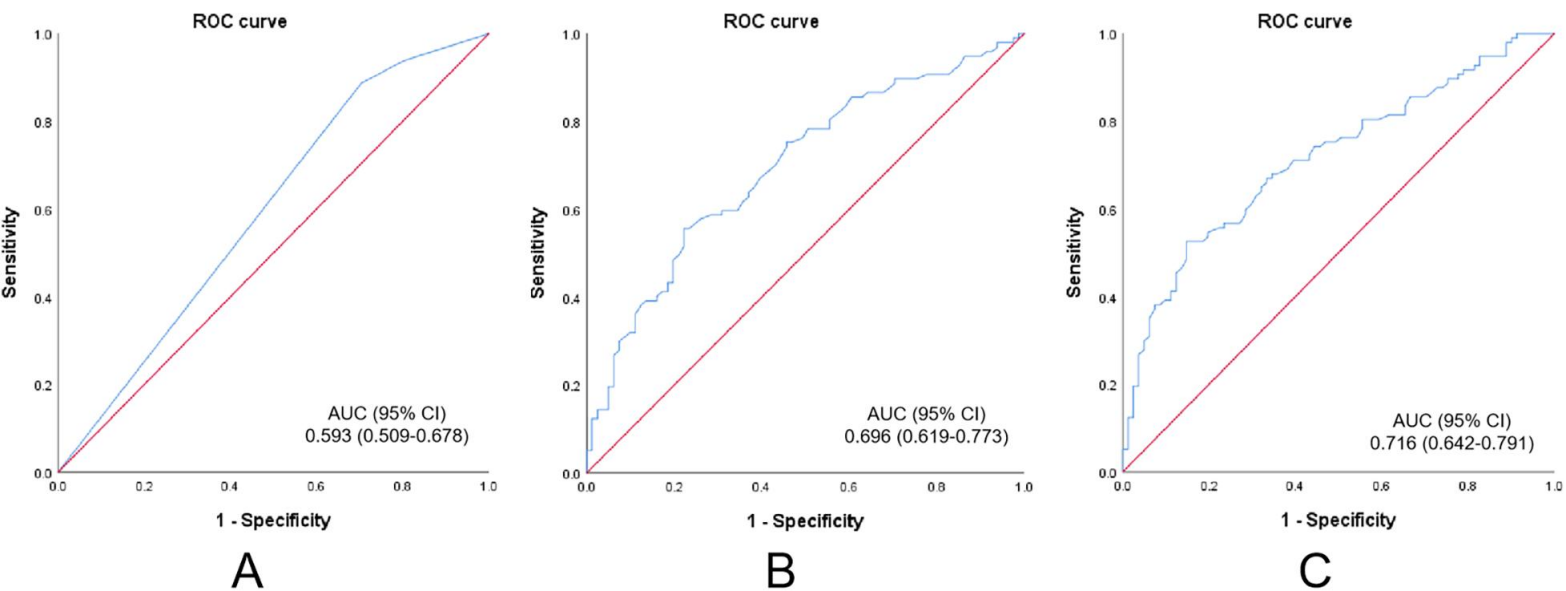
Draw the receiver operating characteristic (ROC) curves of the number of carotid artery plaques **(A)**, the maximum length of carotid artery plaques (mm) **(B)**, and the combination of the number of carotid artery plaques and the maximum length of carotid artery plaques **(C)** for predicting coronary heart disease (CHD) in patients with chronic kidney disease (CKD) and carotid artery plaques. ROC, receiver operating characteristic; AUC, area under the curve; CI, confidence interval.

The predictive performance of carotid plaque number and maximum plaque length for CHD in patients with CKD was evaluated using ROC curve analysis. The area under the curve (AUC) for carotid plaque number was 0.593 (95% CI: 0.509–0.678, *P* = 0.032), with an optimal cutoff value of 2.50. The AUC for maximum plaque length was 0.696 (95% CI: 0.619–0.773, *P* < 0.001), with an optimal cutoff of 8.75 mm. Furthermore, a combined model integrating both plaque number and maximum length yielded an AUC of 0.716 (95% CI: 0.642–0.791, *P* < 0.001), demonstrating enhanced predictive capability. The corresponding ROC curves were presented in Figure 1, and the detailed AUC values, optimal thresholds, and associated sensitivity and specificity measures were summarized in Table 9.

**Table 9:** ROC analysis of statistically significant parameters for predicting CKD-related CHD.

Variables	ROC-AUC (95% CI)	*P* value	Cut-off (sensitivity, specificity)
Carotid artery plaque number	0.593 (0.509–0.678)	0.032	2.50 (0.887, 0.296)
Max Carotid artery plaque length (mm)	0.696 (0.619–0.773)	<0.001	8.75 (0.557, 0.778)
Carotid artery plaque number + Max Carotid artery plaque length (mm)	0.716 (0.642–0.791)	<0.001	(0.526, 0.852)

CKD, chronic kidney disease; CHD, coronary heart disease; ROC, receiver operating characteristic; AUC, area under the curve; CI, confidence interval.

.

## 4 Discussion

In this study, PSM was employed to mitigate non-random selection bias and confounding variables. Subsequently, a cohort of patients with CKD and carotid plaques was selected for investigation. It was confirmed that in CKD patients, the number and maximum length of carotid plaques were correlated with the incidence of CHD. ROC analysis demonstrated that the AUC of the number of carotid plaques was 0.593, with a sensitivity of 88.7% and a specificity of 29.6%. The AUC of the maximum length of carotid plaques was 0.696, with a sensitivity of 55.7% and a specificity of 77.8%. The AUC after combining these two parameters was 0.716, with a sensitivity of 52.6% and a specificity of 85.2%. Consequently, these findings identified two carotid plaque parameters as significant predictors of chronic stable CHD in CKD patients, with combined assessment offering superior predictive value.

Current research predominantly focused on the utility of carotid plaque ultrasound parameters for predicting coronary artery calcification in patients with CKD (17). However, evidence regarding their predictive value for CHD in CKD patients, particularly within the Chinese population, remained limited. Prior study has established that cIMT predicted CHD in end-stage renal disease patients (18). The CKD population in our study mainly comprised early-stage (stage 2) patients. Thus, our study subjects were early-stage (stage 2) CKD patients, a special group. Early identification and intervention of CHD in this group have greater clinical significance. Moreover, although recent research suggested that carotid plaque thickness predicted long-term major adverse cardiovascular events (MACE) in CKD cohorts (19), that study primarily addressed acute events and overlooked stable CHD. This study aimed to bridge this gap by evaluating the predictive capacity of carotid plaque parameters for stable CHD in early-stage CKD patients. This non-invasive assessment strategy was clinically valuable as it circumvented the risk of contrast-induced renal impairment. In summary, while extant literature emphasized the role of carotid plaque metrics in forecasting coronary calcification or adverse events in advanced CKD, investigations into their association with CHD in early-stage CKD were scarce. To our knowledge, this was the first study to demonstrate that carotid plaque number and length were predictive of stable CHD in early-stage CKD patients, thereby offering a safe, non-invasive tool for early risk assessment that could potentially delay disease progression and avoid contrast-associated kidney injury.

Previous studies demonstrated that the carotid plaque score—defined as the total number of plaques detected ultrasonographically in the common carotid artery, bifurcation, and internal carotid artery segments—served as an independent predictor of cardiovascular events and CHD (20, 21), exhibiting superior predictive performance compared to cIMT (22, 23). Total plaque area (TPA) was also significantly associated with multiple cardiovascular risk factors and independently predicted CHD events (24), myocardial infarction (25), and other adverse cardiovascular outcomes (26). The above evidence has indicated that the increase in the number of carotid plaques was associated with an increase in carotid plaque score, which was subsequently linked to the elevation of TPA, and could serve as an important ultrasound indicator for assessing the progression of CHD. In this study, we identified for the first time a significant association between carotid plaque number and stable CHD in patients with early-stage CKD. The underlying mechanism may have involved a direct promotive effect of plaque quantity on TPA accumulation. TPA, as a plaque phenotype closely related to arterial wall remodeling (24), represented a valid marker of systemic atherosclerotic burden. Elevated plaque number and corresponding TPA increase were not only associated with the aggregation of traditional cardiovascular risk factors—such as advanced age, male sex, smoking, dyslipidemia, and hypertension (24, 27)—but also reflected a greater atherosclerotic burden, collectively contributing to the development and progression of CHD. Therefore, our results provided pathophysiological support for the use of carotid plaque number as a biomarker for predicting CHD risk in early-stage CKD patients. Although prior studies had indicated its predictive value, this study was the first to establish the utility of carotid plaque number in stratifying the risk of stable CHD specifically in a CKD population.

Findings from a prior study indicated that the maximum carotid plaque length was superior to traditional risk factors in predicting CHD and the degree of coronary stenosis (28, 29), indicating its potential as a robust predictor of CHD. However, the predictive value of this parameter was not established in patients with early-stage CKD. Our study was the first to confirm that maximum carotid plaque length had significant predictive value for CHD in this population. Although the underlying mechanism remained incompletely elucidated, this parameter—due to its simplicity of measurement and broad applicability—proved to be a valuable tool for predicting CHD and guiding clinical intervention in early-stage CKD patients, thereby potentially improving prognosis.

To our knowledge, this was the first study to demonstrate that carotid ultrasound parameters—specifically, plaque number and maximum plaque length—served as significant predictors of CHD in patients with early-stage CKD. However, several limitations must be noted. First, although our study offered valuable hypotheses, the single-center, retrospective case-control design inherently limits the strength of the evidence. The findings may be influenced by institutional-specific practices and retrospective data collection, potentially compromising their generalizability. Thus, the outcomes presented here must be interpreted as generating hypotheses rather than establishing definitive conclusions, and therefore, they require verification through prospective, multicenter studies. Second, despite the use of PSM to reduce selection bias and control for confounders, the modest sample size may affect the generalizability of our findings; larger cohorts are needed to strengthen the reliability of these observations. Third, although this study adopted a rigorous design and scientific statistical methods, the results showed that the carotid plaque parameters had limited predictive ability for CHD in patients with CKD, with a relatively low AUC value, indicating that their clinical predictive value when used alone was limited. However, after combining the carotid plaque parameters, the predictive model's overall predictive value was improved to an acceptable level. This result may be related to the relatively small sample size of this study. In the future, we will further verify the predictive efficacy of this indicator in CKD patients by increasing the sample size, so as to draw more reliable and generalizable conclusions. Finally, the evaluation of certain ultrasound parameters was limited by institutional data availability, which limited more granular analyses such as integrated plaque scoring. Future studies incorporating more comprehensive ultrasonic phenotypic data may provide deeper mechanistic and diagnostic insights.

## 5 Conclusions

Our study reveals that as the number of carotid plaques and the maximum plaque length increase, the risk of stable CHD in patients with CKD, particularly those in the early stage of CKD, rises significantly. Moreover, the combined use of these two indicators demonstrates a higher predictive value. The carotid plaque ultrasound parameters identified in this study may enable early prediction of CHD in CKD patients, potentially reducing reliance on iodine-containing contrast agents for diagnosis. This approach can facilitate earlier implementation of secondary CHD prevention strategies, thereby reducing long-term cardiovascular events, improving quality of life, and alleviating the associated socioeconomic burden. Prospective, randomized controlled trials are warranted to further validate this association and establish the clinical utility of carotid ultrasound parameters in the management of CKD patients at risk of CHD.

## Data Availability

The original contributions presented in the study are included in the article/Supplementary Material, further inquiries can be directed to the corresponding author.
